# Predictors of Intensive Care Unit Outcomes in Elderly Patients with Acute Respiratory Failure: A Retrospective Cohort Study

**DOI:** 10.3390/jcm14165761

**Published:** 2025-08-14

**Authors:** Nazlıhan Boyacı Dündar, Kamil İnci, İrem Pamuk, Gulbin Aygencel, Melda Turkoglu

**Affiliations:** 1 Division of Intensive Care Medicine, Department of Internal Medicine, Gazi University School of Medicine, Ankara 06500, Turkey; kamilinci@gmail.com (K.İ.); aygencel@hotmail.com (G.A.); meldaturkoglu@yahoo.com.tr (M.T.); 2 Department of Internal Medicine, Gazi University School of Medicine, Ankara 06500, Turkey; irempamuk95@gmail.com

**Keywords:** elderly patients, acute respiratory failure, ICU outcome, requirement of invasive mechanical ventilation support, mortality

## Abstract

**Background/Objectives**: Acute respiratory failure (ARF), a major cause of intensive care unit (ICU) admission in elderly patients, is strongly associated with adverse outcomes. Despite its clinical significance, data on prognostic factors in this patient group remain limited. This study aims to identify key prognostic factors in elderly ICU patients with ARF to guide clinical management. **Methods**: This retrospective cohort study analyzed data from elderly patients (≥65 years) admitted to the tertiary medical ICU of Gazi University Hospital due to ARF between February 2020 and December 2022. Collected data included demographic characteristics, comorbidities, reasons for ICU admission, organ support requirements, and clinical scores. Statistical analyses were performed to identify independent predictors of ICU mortality and invasive mechanical ventilation (IMV) requirement. **Results:** Of 244 patients, the median age was 76 (70–82) years, with a mortality rate of 49.2%. Independent predictors of mortality included higher SOFA scores (OR: 1.316, 95% CI: 1.089–1.590, *p* = 0.005), presence of shock at ICU admission (OR: 2.875, 95% CI: 1.046–7.905, *p* = 0.041), requirement of IMV (OR: 9.415, 95% CI: 3.591–24.679, *p* < 0.001), requirement of renal replacement therapy (RRT) (OR: 3.039, 95% CI: 1.125–8.206, *p* = 0.028), and hypoalbuminemia (OR: 3.647, 95% CI: 1.238–10.742, *p* = 0.019). IMV support was required in 56.9% of patients and was associated with more severe illness, worse oxygenation, and higher ICU mortality (77.6% vs. 11.4%, *p* < 0.001). **Conclusions**: In elderly patients with ARF, ICU mortality was independently associated with organ dysfunctions (higher SOFA scores, presence of shock at ICU admission, requirements of IMV and RRT) and hypoalbuminemia. Our findings highlight the need for individualized risk assessment and targeted supportive strategies in elderly patients with ARF.

## 1. Introduction

Worldwide epidemiological data indicate that adults aged ≥65 years currently account for between one-quarter and one-half of all intensive care unit (ICU) admissions, and this proportion continues to rise in parallel with the aging of the global population [1,2,3]. Acute respiratory failure (ARF) is one of the most common critical conditions encountered in this age group and represents a significant cause of ICU admission [4,5,6,7]. Given these demographic shifts, a better understanding of the outcomes and risk factors associated with ARF in elderly patients has become increasingly important [1,8].

Among elderly ICU patients, multinational prospective studies have shown that outcomes are more strongly influenced by frailty and acute organ dysfunction than by chronological age itself [3,9]. Older adults are particularly vulnerable to adverse consequences due to age-related physiological changes, including diminished respiratory muscle strength, decreased lung compliance, and impaired immune function [10,11,12]. Additionally, multiple comorbidities complicate the presentation and management of ARF in this population [8,10].

Although previous studies have identified factors such as illness severity scores and the need for organ support as important predictors of ICU outcomes, the extent to which these variables affect elderly patients remains insufficiently defined [7,13,14,15]. The role of chronological age as an independent risk factor for mortality remains controversial, with studies yielding conflicting results [15,16,17,18]. Clarifying these issues is essential for optimizing treatment strategies and improving prognostic accuracy in older adults with ARF.

In this study, we aimed to identify key predictors of mortality and adverse outcomes in elderly patients admitted to the ICU with ARF. We sought to enhance risk stratification and guide clinical decision-making in this growing patient population by analyzing clinical, demographic, and physiological variables.

## 2. Materials and Methods

### 2.1. Study Design and Setting

We conducted a retrospective, observational study in the tertiary medical intensive care unit of Gazi University Hospital, an academic center located in Ankara, Turkey. The study period spanned from February 2020 to December 2022, encompassing patients aged 65 and older admitted for ARF.

### 2.2. Inclusion and Exclusion Criteria

Patients were included if they had a primary diagnosis of ARF requiring ICU admission, defined by a need for mechanical ventilation or severe hypoxemia unresponsive to standard oxygen therapy. Exclusion criteria were palliative care status and incomplete medical records.

### 2.3. Data Collection

Demographic data (age, gender), comorbidities (e.g., hypertension, cardiac disorders, malignancy, endocrinological disease, etc.), and ICU admission characteristics were recorded. Clinical data included the etiology of acute respiratory failure (e.g., pneumonia, heart failure, acute exacerbation of chronic respiratory disease, etc.), presence of shock at ICU admission, severity scores such as Acute Physiology and Chronic Health Evaluation II (APACHE II) and Sequential Organ Failure Assessment (SOFA), and the Risk, Injury, Failure, Loss, and End-stage renal disease classification for acute kidney injury (RIFLE classification). Within the scope of this study, the category of “decompensated heart failure” primarily included patients with acute decompensation of chronic heart failure (CHF), pulmonary edema, right ventricular failure, or cardiogenic shock. Patients admitted due to acute coronary syndromes were typically managed in the coronary ICU (a separate unit from the medical ICU in our institution) and were not included in this cohort. Among patients presenting with shock, subclassification was performed based on clinical and laboratory findings as septic shock, cardiogenic shock, anaphylactic shock, and hemorrhagic shock. The level of consciousness was assessed by the Glasgow Coma Scale (GCS), and oxygenation parameters such as peripheral oxygen saturation (SpO_2_), fraction of inspired oxygen (FiO_2_), and the ratio of partial arterial oxygen pressure to FiO_2_ (P/F ratio) were documented. In addition to established oxygenation parameters, the Respiratory Rate–Oxygenation Index (ROX index) was calculated for all patients to assess its association with invasive mechanical ventilation (IMV) requirement in elderly patients with ARF, considering its reported use in various patient groups receiving oxygen support therapies beyond high-flow nasal oxygen (HFNO) [19]. In cases of pneumonia, data on the identified etiologic pathogens, including bacterial, viral, and fungal, and cases with no isolated microorganism, were also collected, along with a documented history of aspiration. Laboratory values [e.g., C-reactive protein (CRP), procalcitonin, albumin, lactate, renal and liver function tests] and blood gas analyses obtained on the day of ICU admission were documented. Treatment-related factors were collected, including non-invasive ventilation (NIV), IMV, HFNO, vasopressors, renal replacement therapy (RRT), and nutritional support. However, detailed data regarding the timing and duration of these interventions were not recorded, as they were not directly related to the study’s primary outcome of ICU mortality. Additionally, the duration of hospital stay before ICU admission and the length of ICU stay were also recorded.

### 2.4. Outcome Measures

The primary outcome was ICU mortality. The secondary outcome was identifying clinical and physiological factors associated with the requirement for IMV.

### 2.5. Statistical Analysis

Continuous variables were presented as mean ± standard deviation (SD) or median with interquartile range (IQR) [25th–75th percentile], based on data distribution assessed by the Kolmogorov–Smirnov test. Categorical variables were expressed as counts and percentages. Group comparisons between survivors and non-survivors and between patients with and without IMV support were performed using the chi-square test for categorical variables and either the independent samples *t*-test or Mann–Whitney U test for continuous variables, as appropriate. Univariate logistic regression analyses were conducted to identify factors associated with ICU mortality and IMV requirement. Variables with a *p*-value < 0.10 in univariate analysis were included in the multivariate logistic regression model to identify independent predictors. A two-sided *p*-value < 0.05 was considered statistically significant. All analyses were performed using IBM SPSS Statistics [Version 29.0.2.0 (20)].

## 3. Results

During the study period, a total of 244 geriatric patients with a median age of 76 years (IQR: 71–82) were admitted to the ICU due to ARF. Of these, 61.5% were male. Pneumonia was the most common etiology (62.7%), followed by decompensated heart failure (38.9%) and acute exacerbations of chronic respiratory diseases (34.8%). The most frequently observed comorbidities were cardiac disorders (73.7%), hypertension (63.8%), and pulmonary diseases (52.3%). Detailed baseline characteristics according to ICU outcome are presented in Table 1.

### 3.1. Patient Characteristics

At ICU admission, the median APACHE II and SOFA scores were 20 [16–27] and 6 [3–9], respectively. Respiratory parameters measured at ICU admission revealed a median P/F ratio of 163 [116–232] and a median ROX index of 9.56 [6.53–13.5]. Among patients diagnosed with pneumonia, Gram-negative microorganisms were identified in 16% of cases, while Gram-positive bacteria were detected in 5.7%; no causative pathogen could be isolated in 28.6% of the patients. A history of aspiration was documented in 15.2% of the total cohort. IMV was required in 56.9% of patients, while 32.9% received NIV and 7.9% were supported with HFNO. Additionally, 36.2% of patients presented with shock on admission. Among these, septic shock was the predominant type, while two patients presented with cardiogenic shock, one with anaphylactic shock, one with hemorrhagic shock, and two with both septic and cardiogenic shock. The clinical characteristics according to ICU outcome are summarized in Table 1.

### 3.2. ICU Outcomes and Mortality-Associated Factors

The overall ICU mortality rate was 49.2% (120). Compared with survivors, non-survivors had significantly higher APACHE II (25 [18–30] vs. 18 [14–22], *p* < 0.001) and SOFA scores (8 [5–11] vs. 4 [2–6], *p* < 0.001), and lower GCS scores (10 [6–15] vs. 14 [12–15], *p* < 0.001) (Table 1). Survivors had significantly better oxygenation parameters, with higher P/F ratios (173 [131–240] vs. 154 [95–220], *p* < 0.001) and ROX indices (11.11 [7.75–14.6] vs. 8.6 [5–12.34], *p* = 0.039) than non-survivors (Table 1). Non-survivors were more likely to require IMV (90.8% vs. 24.4%, *p* < 0.001), vasopressors (shock at admission: 62.5% vs. 10.6%, *p* < 0.001), and RRT (54.6% vs. 16.3%, *p* < 0.001). They also had higher rates of pneumonia as a reason for ARF (77.5% vs. 48.4%, *p* < 0.001) and malignancy (50% vs. 23.6%, *p* < 0.001) (Table 1). Regarding pneumonia etiology, gram-negative microorganisms were more frequently isolated in non-survivors than survivors (8.3% vs. 3.2%, *p* = 0.017). Laboratory findings showed that non-survivors had significantly higher lactate, CRP, procalcitonin, and liver enzymes and more frequent hypoalbuminemia (84% vs. 53.2%, *p* < 0.001) (Table 2).

On multivariate analysis, independent predictors of ICU mortality included IMV requirement (OR: 9.415, 95% CI: 3.591–24.679, *p* < 0.001), presence of shock at ICU admission (OR: 2.875, 95% CI: 1.046–7.905, *p* = 0.041), RRT requirement (OR: 3.039, 95% CI: 1.125–8.206, *p* = 0.028), SOFA score (OR: 1.316, 95% CI: 1.089–1.590, *p* = 0.005), and hypoalbuminemia (OR: 3.647, 95% CI: 1.238–10.742, *p* = 0.019) (Table 3). While the *p*-value for the APACHE II score was 0.049, the upper bound of its 95% confidence interval was exactly 1.000 (OR: 0.921; 95% CI: 0.849–1.000), indicating a borderline association with mortality that did not meet the criteria for robust statistical significance in our model.

### 3.3. Requirement of IMV and Related Factors

Among the cohort, 139 patients (56.9%) required IMV support. Compared with those who did not receive IMV support, patients supported with IMV were younger (75 [70–81] vs. 78 [73–84], p = 0.042), had higher APACHE II (25 [18–30] vs. 17 [14–20], *p* < 0.001) and SOFA scores (8 [5–11] vs. 4 [2–6], *p* < 0.001), and lower GCS scores (9 [6–14] vs. 15 [13–15], *p* < 0.001) (Table 4). IMV-supported patients also had longer hospital stays before ICU (4 [1–13] vs. 1 [0–3], *p* < 0.001), higher rates of pneumonia (76.9% vs. 43.8%, *p* < 0.001), and lower rates of heart failure (28.8% vs. 52.4%, *p* < 0.001) as a reason for ARF (Table 4). Patients requiring IMV support exhibited poorer oxygenation profiles, as reflected by lower P/F ratios (153 [95–219] vs. 188 [140–240], *p* < 0.001) and ROX indices (8.82 [5.06–12.44] vs. 11.06 [7.3–15.1], *p* < 0.001) compared with those not requiring IMV. Additionally, viral pathogens (10.1% vs. 2.9%, *p* = 0.027) and cases without an identified causative microorganism (34.5% vs. 20.0%, *p* = 0.011) were more frequently observed among the IMV-supported group. Hypoalbuminemia was also more prevalent among IMV-supported patients (81.3% vs. 50.5%, *p* < 0.001), as were elevated CRP, procalcitonin, and lactate levels (Table 5).

Multivariate logistic regression identified pneumonia as a reason for ARF (OR: 2.575, 95% CI: 1.083–6.120, *p* = 0.032), ROX index (OR: 0.908, 95% CI: 0.839–0.983, *p* = 0.017), and hypoalbuminemia (OR: 4.115, 95% CI: 1.743–9.719, *p* = 0.001) as independent predictors of IMV requirement. Other variables, including age, gender, P/F ratio, and inflammatory markers (CRP, procalcitonin), were not independently associated with IMV requirement in the adjusted model (Table 6).

## 4. Discussion

Our analysis provides several key insights into the determinants of mortality and mechanical ventilation requirements in elderly patients admitted to the ICU with ARF. Rather than chronological age, outcomes were independently associated with indices of acute physiological derangement, including higher SOFA scores, presence of shock, hypoalbuminemia, and the need for organ support. The requirement of IMV, which was a factor independently associated with ICU mortality in our cohort, was also related to the underlying etiology of ARF, impaired oxygenation indices, and hypoalbuminemia, underscoring the importance of comprehensive clinical assessment beyond age-based risk stratification.

The ICU mortality rate in our cohort was 49.2%, which is notably higher than hospital mortality rates (12.7–27%) reported for elderly patients admitted with respiratory failure to the emergency department or geriatric units [20,21]. In a recent large-scale national study by Hazar et al., which evaluated patients hospitalized due to acute respiratory disease, inpatient mortality was reported at 4.5%. In contrast, ICU and palliative care unit mortality were significantly higher, reaching 34.9% and 40.9%, more closely aligning with our findings [16]. These findings reflect the distinct clinical spectra and disease severities of elderly patients progressing to critical illness and underscore the heterogeneity of ARF etiologies across care settings. Notably, while previous studies conducted in emergency and geriatric settings frequently identified congestive heart failure and acute-on-chronic respiratory failure [e.g., exacerbation of chronic obstructive pulmonary disease (COPD)] as the leading causes of ARF, pneumonia was the most common etiology in our cohort, accounting for 62% of cases [11,20,21]. In recent studies focusing on elderly patients with ARF secondary to coronavirus disease 2019 (COVID-19) pneumonia, a notably high rate of 66.9–65.5% was reported [15,22]. According to comparative data, both congestive heart failure and COPD-related exacerbations are associated with substantially lower short-term mortality than pneumonia [11,21,23]. Thus, the predominance of pneumonia as the leading cause of ARF in our study population may partially account for the observed higher ICU mortality, further emphasizing the prognostic significance of ARF etiology in elderly patients.

Our multivariate model underscores that indices of acute physiological derangement, including higher SOFA scores, presence of shock, the need for organ support, and hypoalbuminemia, rather than chronological age, drive short-term prognosis in elderly patients with ARF. Consistent with a recent study in nonagenarian ICU patients, where day 1 initiation of IMV and dialysis emerged as the strongest independent predictors of 1-year mortality, our findings similarly suggest that timely initiation of life-sustaining therapies, particularly IMV and RRT, is closely linked with adverse outcomes and should be prioritized over age-based thresholds in critical care decision-making [7]. The presence of shock at ICU admission and each incremental increase in SOFA score further align with the systematic review by Vallet et al., which identified illness severity scores and the need for ventilatory support as the most reliable ICU mortality predictors among elderly patients [8]. In our cohort, although the APACHE II score demonstrated a borderline association with ICU mortality (OR: 0.921; 95% CI: 0.849–1.000; *p* = 0.049), its prognostic value was limited compared with the SOFA score. This finding is consistent with the study by Qiao et al., in which both APACHE II and SOFA scores were shown to be useful prognostic tools, but the maximum SOFA score demonstrated superior discriminative ability (AUC = 0.98) compared with APACHE II (AUC = 0.76) [24]. The ability of SOFA to objectively reflect the extent of six-organ dysfunction, regardless of age, makes it a particularly robust prognostic tool in geriatric critical illness [14].

Of particular interest is hypoalbuminemia, observed in over 80% of non-survivors in our cohort, which emerged as a strong but often under-recognized predictor of mortality. A recent study previously demonstrated a nearly two-fold increase in mortality risk among septic older adults when serum albumin fell below 2.85 g/dL, a finding that closely parallels our results [25]. Moreover, in our recently published study evaluating critically ill elderly patients with hypophosphatemia, low albumin once again emerged as an independent factor associated with ICU mortality [26]. Beyond reflecting systemic inflammation, hypoalbuminemia often indicates underlying sarcopenia and malnutrition; these conditions are highly prevalent in frail elderly individuals and are known to impair immune function, prolong recovery, and worsen clinical course [26,27,28]. When used with SOFA, serum albumin may provide a pragmatic bedside marker that captures overall disease burden and nutritional reserve and may offer a practical, age-independent tool for early risk stratification.

In constructing the multivariate model for IMV requirement, we deliberately excluded disease severity scores (e.g., APACHE II, SOFA, GCS) and downstream organ-support variables such as the presence of shock or the requirement for RRT, as these parameters primarily reflect the physiological consequences of acute deterioration rather than serve as direct predictors of IMV [14]. Within this framework, pneumonia as the underlying etiology, a lower ROX index, and hypoalbuminemia were identified as independent factors associated with IMV requirement in our cohort, each plausibly capturing the earlier trajectory of ARF. Although published data specific to elderly critically ill patients with ARF are insufficient, several well-established observations support our findings in this context. First, the paradoxical finding that IMV was more common among the relatively younger elderly patients mirrors prior reports in which the very old were less frequently intubated, presumably owing to a more conservative treatment paradigm or end-of-life care preferences in advanced age [29,30]. Second, the dominance of pneumonia, together with the prolonged hospital stay before ICU admission, raises the likelihood of hospital-acquired or health-care–associated pneumonias, conditions consistently associated with poorer outcomes in older adults [31,32]. Third, the ROX index, a simple bedside ratio incorporating SpO_2_/FiO_2_ and respiratory rate, has consistently been used to predict impending intubation in patients with hypoxemic pneumonia, particularly in the context of HFNO therapy and, more recently, in COVID-19 cohorts [33,34,35,36]. However, beyond its established role in HFNO settings, our findings suggest that the ROX index may also serve as a practical and readily calculable tool for identifying clinical deterioration and anticipating IMV requirements in elderly patients with ARF, regardless of the type of oxygen therapy administered. This observation is in line with the study by Reyes et al., who retrospectively analyzed 895 hospitalized patients with community-acquired pneumonia, in whom HFNC was not administered, and reported that the ROX index was a good predictor of IMV, with an area under the ROC curve of 0.733 (95% CI: 0.671–0.795, *p* < 0.001) [19]. Finally, hypoalbuminemia, both a negative acute-phase reactant and a marker of chronic malnutrition and sarcopenia in elderly individuals, reaffirmed its prognostic relevance [26,27,28]. Its predictive role in our cohort is consistent with previous evidence linking low albumin levels to adverse clinical outcomes across various disease subgroups [25,26,28,37]. These findings suggest that, even among elderly patients, the need for IMV is determined more by biological factors, such as infection type, the severity of hypoxemia, and systemic inflammation, and easily measurable physiological indices, rather than by chronological age alone [22]. This highlights the importance of early, physiology-driven clinical monitoring and nutritional optimization in this vulnerable population.

Strengths of this study include its focus on a well-defined and clinically relevant population, elderly patients admitted to the ICU with ARF, and the simultaneous evaluation of mortality and IMV requirements. Including pragmatic, bedside-applicable variables such as the ROX index and serum albumin enhances the clinical utility of the findings. Furthermore, the study offers insight into decision-making patterns and physiological trajectories that are not easily captured in broader and mixed population datasets.

Nonetheless, our study has several limitations that should be acknowledged. First, its single-center and retrospective design limits generalizability and precludes causal inference. Second, the relatively small sample size may affect the stability of multivariate estimates. Third, variables such as care goal preferences, functional status, and frailty scores, which are known to influence ICU decision-making in the elderly population, were not systematically captured and may have introduced residual confounding. In addition, due to the limited sample size and the exploratory nature of our analysis, we were unable to adjust for all potential confounding variables in the multivariable model. Therefore, associations identified in this study should be interpreted with caution, as unmeasured or unadjusted confounders may have influenced the observed relationships. Fourth, due to the structure of the ICU database, specific subtypes of cardiac disorders could not be separately analyzed, although these entities may differ in their underlying pathophysiology and associated prognosis, potentially influencing the observed outcomes. Finally, long-term outcomes after ICU discharge were not assessed.

## 5. Conclusions

This study demonstrates that in elderly ICU patients with acute respiratory failure, short-term outcomes such as mortality and the need for IMV are primarily determined by acute physiological deterioration and disease severity, rather than chronological age. The combined use of the SOFA score and serum albumin level provides a practical and accessible tool for early risk stratification in this population. These findings emphasize the importance of timely physiological evaluation, integration of age-specific considerations, and individualized management to improve outcomes in critically ill elderly patients with ARF.

## Figures and Tables

**Table 1:** Baseline characteristics and ICU-related data in the study patients according to ICU outcome.

	All Patients (n = 244)	Survivors (n = 124)	Non-Survivors (n = 120)	*p*
Age *	76 [71–82]	78 [73–83]	75 [70–82]	0.097
**Gender, n (%)**				0.102
Female	94 (38.5)	54 (43.4)	40 (33.3)	
Male	150 (61.5)	70 (56.6)	80 (66.7)	
APACHE II Score *	20 [16–27]	18 [14–22]	25 [18–30]	**<0.001**
SOFA Score *	6 [3–9]	4 [2–6]	8 [5–11]	**<0.001**
Glasgow Coma Scale *	13 [8–15]	14 [12–15]	10 [6–15]	**<0.001**
Length of ICU stay (day) *	8 [4–18]	7 [3–11]	10 [5–23]	**0.007**
Length of hospital stay before ICU (day) *	2 [0–8]	1 [0–3]	4 [1–14]	**<0.001**
Respiratory Parameters *				
Respiratory rate (/min)	23 [20–26]	22 [20–26]	23 [20–26]	0.437
FiO_2_ (%)	41 [31–60]	40 [30–50]	50 [37–70]	**<0.001**
SpO_2_ (%)	94 [91–97]	95 [92–97]	93 [90–97]	0.137
P/F ratio	163 [116–232]	173 [131–240]	154 [95–220]	**<0.001**
ROX index	9.56 [6.53–13.5]	11.11 [7.75–14.6]	8.6 [5–12.34]	**0.039**
**Reasons for Respiratory Failure, n (%)**				
Pneumonia	153 (62.7)	60 (48.4)	93 (77.5)	**<0.001**
Decompensated Heart Failure	95 (38.9)	59 (47.6)	36 (30.0)	**0.005**
Acute Exacerbation of CRD	85 (34.8)	50 (40.3)	35 (29.2)	0.067
Extrapulmonary Causes	33 (13.5)	16 (12.9)	17 (14.2)	0.773
Malignancy	21 (8.6)	4 (3.2)	17 (14.2)	**0.002**
Pulmonary Embolism	20 (8.2)	9 (7.3)	11 (9.2)	0.587
Airway compromise due to impaired consciousness	13 (5.3)	6 (4.8)	7 (5.8)	0.729
**Pneumonia Etiologies, n (%)**				
Gram (−) microorganism	39 (16)	13 (10.5)	26 (21.7)	**0.017**
Gram (+) microorganism	14 (5.7)	4 (3.2)	10 (8.3)	0.086
Viral	17 (7.0)	9 (7.3)	8 (6.7)	0.856
Fungal	17 (7.0)	6 (4.8)	11 (9.2)	0.184
No isolated pathogen	70 (28.6)	28 (21.8)	42 (35)	**0.022**
History of Aspiration, n (%)	37 (15.2)	17 (13.7)	20 (16.7)	0.520
Presence of shock at ICU admission, n (%)	88 (36.2)	13 (10.6)	75 (62.5)	**<0.001**
Presence of sepsis at ICU admission, n (%)	120 (49.2)	33 (26.6)	87 (72.5)	**<0.001**
**Comorbidities, n (%)**				
Cardiac disorders	179 (73.7)	89 (72.4)	90 (75.0)	0.640
Hypertension	155 (63.8)	87 (70.7)	68 (56.7)	**0.023**
Pulmonary disease	127 (52.3)	70 (56.9)	57 (47.5)	0.142
Endocrinological disease	82 (33.9)	43 (35.2)	39 (32.5)	0.652
Malignancy	89 (36.6)	29 (23.6)	60 (50.0)	**<0.001**
Chronic renal disease	75 (30.9)	38 (30.1)	37 (31.7)	0.789
Neurological disease	60 (24.7)	37 (30.1)	23 (19.2)	**0.049**
Gastroenterologic disorders	12 (4.9)	4 (3.3)	8 (6.7)	0.219
Rheumatological disease	5 (2.1)	3 (2.4)	2 (1.7)	1.00
**Requirement of respiratory support, n (%)**				
Invasive mechanical ventilation	139 (56.9)	30 (24.4)	109 (90.8)	**<0.001**
Non-invasive mechanical ventilation	80 (32.9)	52 (42.3)	28 (23.3)	**0.002**
HFNO	19 (7.9)	10 (8.1)	9 (7.6)	0.870
Nasal oxygen therapy	53 (23.4)	48 (38.7)	9 (7.5)	**<0.001**
**RIFLE stage at ICU admission, n (%)**				
Risk	63 (25.9)	31 (25.2)	32 (26.7)	0.795
Injury	28 (11.5)	13 (10.6)	15 (12.5)	0.637
Failure	42 (17.3)	14 (11.4)	28 (23.3)	**0.014**
Loss	5 (2.1)	4 (3.3)	1 (0.8)	0.370
End-stage	19 (7.8)	5 (4.1)	14 (11.7)	**0.027**
**Requirement of RRT, n (%)**	85 (35.1)	20 (16.3)	65 (54.6)	**<0.001**
Intermittent hemodialysis	59 (24.4)	16 (13.0)	43 (36.1)	**<0.001**
CRRT	49 (20.2)	4 (3.3)	45 (37.8)	**<0.001**
**Nutritional support, n (%)**				
Parenteral	19 (7.9)	6 (4.9)	13 (10.9)	0.080
Enteral	164 (67.5)	73 (59.3)	91 (75.8)	**0.006**

* median [25th percentile–75th percentile]. ICU: Intensive Care Unit, APACHE: Acute Physiology and Chronic Health Evaluation, SOFA: Sequential Organ Failure Assessment, FiO_2_: Fraction of Inspired Oxygen, SpO_2_: Peripheral Capillary Oxygen Saturation, P/F: Partial Pressure of Arterial Oxygen to Fraction of Inspired Oxygen, ROX: Respiratory Rate–Oxygenation Index, CRD: Chronic Respiratory Disease, HFNO: High Flow Nasal Oxygen, RIFLE: Risk, Injury, Failure, Loss, End-stage Classification for Renal Failure, RRT: Renal Replacement Therapy, CRRT: Continuous Renal Replacement Therapy.

**Table 2:** Baseline laboratory findings in the study patients according to ICU outcome.

	All Patients (n = 244)	Survivors (n = 124)	Non-Survivors (n = 120)	***p***
Blood Urea Nitrogen (mg/dL) *	42 [26–61]	34 [23–58]	47 [32–66]	**0.003**
Creatinine (mg/dL) *	1.32 [0.85–2.38]	1.18 [0.83–2.24]	1.6 [0.87–2.8]	0.130
Sodium (mEq/L) *	139 [135–142]	139 [136–142]	138 [134–142]	0.121
Chloride (mEq/L) *	102 [96–107]	101 [98–107]	103 [97–107]	0.865
Potassium (mEq/L) *	4.1 [3.6–4.6]	4.0 [3.6–4.6]	4.1 [3.6–4.8]	0.233
Calcium (mg/dL) *	9.1 [8.4–9.6]	9.1 [8.5–9.6]	9.2 [8.3–9.7]	0.880
Phosphorus (mg/dL) *	3.5 [2.8–4.8]	3.4 [2.8–4.5]	3.7 [2.8–5.4]	0.229
Magnesium (mg/dL) *	1.9 [1.8–2.2]	1.9 [1.7–2.1]	2.0 [1.8–2.2]	0.065
Alanine transaminase (U/L) *	21 [12–42]	16 [11–31]	26 [14–55]	**<0.001**
Aspartate transaminase (U/L) *	28 [17–53]	24 [15–36]	37 [21–70]	**<0.001**
Total Bilirubin (mg/dL) *	0.86 [0.6–1.34]	0.78 [0.54–1.15]	0.99 [0.64–1.58]	**0.003**
Albumin (g/dL) *	2.6 [2.3–3.1]	2.9 [2.4–3.3]	2.5 [2.2–2.8]	**<0.001**
Hypoalbuminemia, n (%)	166 (68.3)	66 (53.2)	100 (84)	**<0.001**
D-dimer *	2.9 [1.6–6.7]	2.5 [1.6–4.9]	4.2 [1.6–10.3]	0.061
proBNP *	7300 [2900–18,500]	6040 [2300–18,000]	7900 [3500–34,000]	0.273
Arterial blood gas sampling *				
pH	7.36 [7.30–7.44]	7.39 [7.32–7.45]	7.35 [7.26–7.42]	**0.004**
PCO_2_ (mmHg)	38 [31–49]	39 [32–52]	38 [31–46]	0.271
PO_2_ (mmHg)	71 [61–87]	69 [59–84]	75 [64–91]	0.091
HCO_3_ (mEq/L)	22.5 [18.6–27.5]	25.2 [19.8–28.8]	20.6 [16.1–25.2]	**<0.001**
Lactate (mmol/L)	1.7 [1.2–2.5]	1.5 [1.0–2.0]	2.1 [1.5–3.0]	**<0.001**
WBC (×10^3^/µL) *	10.7 [7.5–17.7]	10.5 [7.7–14.9]	12.1 [7.0–16.8]	0.798
NLR *	10.6 [5.8–21.2]	10.2 [5.6–20.1]	12.1 [7.0–18.8]	0.202
C-reactive protein (mg/L) *	98 [38–169]	83 [28–146]	124 [71–200]	**<0.001**
Procalcitonin (ng/mL) *	0.73 [0.26–2.84]	0.42 [0.16–2.13]	1.22 [0.36–5.2]	**<0.001**

* median [25th percentile–75th percentile]. proBNP: Pro–Brain-type Natriuretic Peptide, PCO_2_: Partial Pressure of Carbon Dioxide, PO_2_: Partial Pressure of Oxygen, HCO_3_: Bicarbonate, WBC: White Blood Cell Count, NLR: Neutrophil-to-Lymphocyte Ratio.

**Table 3:** Multivariate analysis for independent risk factors of ICU mortality in elderly critically ill patients with acute respiratory failure.

	Adjusted OR [95% CI]	*p*
APACHE II Score	0.921 [0.849–1.000]	0.049
SOFA Score	1.316 [1.089–1.590]	**0.005**
Pneumonia as a reason for respiratory failure	1.523 [0.559–4.150]	0.410
Malignancy as a reason for respiratory failure	1.792 [0.293–10.953]	0.528
Presence of shock at ICU admission	2.875 [1.046–7.905]	**0.041**
Requirement of IMV	9.415 [3.591–24.679]	**<0.001**
Requirement of RRT	3.039 [1.125–8.206]	**0.028**
ROX index	0.950 [0.880–1.025]	0.187
Pneumonia due to Gram-negative bacteria	1.166 [0.368–3.698]	0.794
CRP	1.002 [0.998–1.006]	0.373
Length of hospital stay before ICU	1.015 [0.973–1.059]	0.476
Hypoalbuminemia	3.647 [1.238–10.742]	**0.019**

APACHE: Acute Physiology and Chronic Health Evaluation, SOFA: Sequential Organ Failure Assessment, ICU: Intensive Care Unit, IMV: Invasive Mechanical Ventilation, RRT: Renal Replacement Therapy, ROX: Respiratory Rate–Oxygenation Index, CRP: C-reactive Protein.

**Table 4:** Baseline characteristics and ICU-related data in the study patients according to the requirement of invasive mechanical ventilation.

	All Patients n = 244	IMV (−) Group n = 105	IMV (+) Group n = 139	*p*
Age *	76 [71–82]	78 [73–84]	75 [70–81]	**0.042**
**Gender, n (%)**				**0.050**
Female	94 (38.5)	48 (45.7)	46 (33.1)	
Male	150 (61.5)	57 (54.3)	93 (66.9)	
APACHE II Score *	20 [16–27]	17 [14–20]	25 [18–30]	**<0.001**
SOFA Score *	6 [3–9]	4 [2–6]	8 [5–11]	**<0.001**
Glasgow Coma Scale *	13 [8–15]	15 [13–15]	9 [6–14]	**<0.001**
Length of ICU stay (day) *	8 [4–18]	6 [3–8]	13 [5–27]	**<0.001**
Length of hospital stay before ICU (day) *	2 [0–8]	1 [0–3]	4 [1–13]	**<0.001**
Respiratory Parameters *				
Respiratory rate (/min)	23 [20–26]	23 [20–26]	23 [20–26]	0.990
FiO_2_ (%)	41 [31–60]	37 [30–50]	50 [37–68]	**<0.001**
SpO_2_ (%)	94 [91–97]	94 [92–97]	94 [91–97]	0.868
P/F ratio	163 [116–232]	188 [140–240]	153 [95–219]	**<0.001**
ROX index	9.56 [6.53–13.5]	11.06 [7.3–15.1]	8.82 [5.06–12.44]	**<0.001**
**Reasons for Respiratory Failure, n (%)**				
Pneumonia	153 (62.7)	46 (43.8)	107 (76.9)	**<0.001**
Decompensated Heart Failure	95 (38.9)	55 (52.4)	40 (28.8)	**<0.001**
Acute Exacerbation of CRD	85 (34.8)	43 (41.0)	42 (30.2)	0.866
Extrapulmonary Causes	33 (13.5)	12 (11.4)	21 (15.1)	0.393
Malignancy	21 (8.6)	4 (3.8)	17 (12.2)	**0.019**
Pulmonary Embolism	20 (8.2)	9 (8.6)	11 (7.9)	0.089
Airway compromise due to impaired consciousness	13 (5.3)	5 (4.8)	8 (5.8)	0.722
**Pneumonia Etiologies, n (%)**				
Gram (-) microorganism	39 (16)	12 (11.4)	27 (19.4)	0.087
Gram (+) microorganism	14 (5.7)	3 (2.9)	11 (7.9)	0.90
Viral	17 (7.0)	3 (2.9)	14 (10.1)	**0.027**
Fungal	17 (7.0)	5 (4.8)	12 (8.6)	0.234
No isolated pathogen	70 (28.6)	21 (20)	49 (35.3)	**0.011**
History of Aspiration, n (%)	37 (15.2)	16 (15.2)	21 (15.1)	0.996
Presence of shock at ICU admission, n (%)	88 (36.2)	8 (7.6)	80 (57.6)	**<0.001**
Presence of sepsis at ICU admission, n (%)	120 (49.2)	22 (21.0)	87 (72.5)	**<0.001**
**Comorbidities, n (%)**				
Cardiac disorders	179 (73.7)	80 (76.2)	99 (71.2)	0.435
Hypertension	155 (63.8)	73 (69.5)	82 (59.0)	0.105
Pulmonary disease	127 (52.3)	60 (57.1)	67 (48.2)	0.184
Endocrinological disease	82 (33.9)	28 (26.7)	61 (43.9)	**0.005**
Malignancy	89 (36.6)	35 (33.7)	47 (33.8)	0.948
Chronic renal disease	75 (30.9)	38 (36.2)	37 (26.6)	0.117
Neurological disease	60 (24.7)	29 (27.6)	31 (22.3)	0.356
Gastroenterologic disorders	12 (4.9)	5 (4.8)	7 (5.1)	0.912
Rheumatological disease	5 (2.1)	3 (2.9)	2 (1.4)	0.655
**Requirement of respiratory support, n (%)**				
Non-invasive mechanical ventilation	80 (32.9)	44 (41.9)	36 (25.9)	**0.009**
HFNO	19 (7.9)	10 (9.5)	9 (6.5)	0.397
**RIFLE stage at ICU admission, n (%)**				
Risk	63 (25.9)	30 (28.6)	33 (23.7)	0.412
Injury	28 (11.5)	11 (10.5)	17 (12.2)	0.656
Failure	42 (17.3)	12 (11.4)	30 (21.6)	0.035
Loss	5 (2.1)	3 (2.9)	2 (1.4)	0.655
End-stage	19 (7.8)	5 (4.8)	14 (10.1)	0.122
**Requirement of RRT, n (%)**	85 (35.1)	14 (13.3)	71 (51.1)	**<0.001**
Intermittent hemodialysis	59 (24.4)	13 (12.4)	46 (33.1)	**<0.001**
CRRT	49 (20.2)	1 (1)	48 (34.5)	**<0.001**
**Nutritional support, n (%)**				
Parenteral	19 (7.9)	4 (3.8)	15 (10.8)	**0.041**
Enteral	164 (67.5)	54 (51.4)	110 (79.1)	**<0.001**

* median [25th percentile–75th percentile]. ICU: Intensive Care Unit, APACHE: Acute Physiology and Chronic Health Evaluation, SOFA: Sequential Organ Failure Assessment, FiO_2_: Fraction of Inspired Oxygen, SpO_2_: Peripheral Capillary Oxygen Saturation, P/F: Partial Pressure of Arterial Oxygen to Fraction of Inspired Oxygen, ROX: Respiratory Rate–Oxygenation Index, CRD: Chronic Respiratory Disease, HFNO: High Flow Nasal Oxygen, RIFLE: Risk, Injury, Failure, Loss, End-stage Classification for Renal Failure, RRT: Renal Replacement Therapy, CRRT: Continuous Renal Replacement Therapy.

**Table 5:** Baseline laboratory findings in study patients according to the requirement of invasive mechanical ventilation.

	All Patients n = 244	IMV (−) Group n = 105	IMV (+) Group n = 139	***p***
Blood Urea Nitrogen (mg/dL)	42 [26–61]	36 [24–59]	45 [29–65]	0.084
Creatinine (mg/dL)	1.32 [0.85–2.38]	1.29 [0.92–2.25]	1.42 [0.74–2.73]	0.938
Sodium (mEq/L)	139 [135–142]	139 [136–142]	139 [135–142]	0.913
Chloride (mEq/L)	102 [96–107]	101 [97–107]	103 [98–108]	0.508
Potassium (mEq/L)	4.1 [3.6–4.6]	4.1 [3.6–4.7]	4.0 [3.5–4.6]	0.652
Calcium (mg/dL)	9.1 [8.4–9.6]	9.1 [8.5–9.5]	9.2 [8.3–9.6]	0.857
Phosphorus (mg/dL)	3.5 [2.8–4.8]	3.5 [3.0–4.6]	3.45 [2.6–5.1]	0.982
Magnesium (mg/dL)	1.9 [1.8–2.2]	1.9 [1.7–2.2]	2 [1.8–2.2]	0.289
Alanine transaminase (U/L)	21 [12–42]	16 [11–30]	25 [13–55]	**0.003**
Aspartate transaminase (U/L)	28 [17–53]	23 [13–36]	33 [19–67]	**<0.001**
Total Bilirubin (mg/dL)	0.86 [0.6–1.34]	1.23 [1.12–1.45]	1.34 [1.2–1.61]	**0.007**
Albumin (g/dL)	2.6 [2.3–3.1]	2.9 [2.5–3.4]	2.5 [2.2–2.8]	**<0.001**
Hypoalbuminemia, n (%)	166 (68.3)	53 (50.5)	113 (81.3)	**<0.001**
D-dimer	2.9 [1.6–6.7]	2.2 [1.2–5.0]	3.3 [1.8–9.8]	0.059
proBNP	7300 [2900–18,500]	6000 [2800–16,000]	7900 [3500–28,000]	0.266
Arterial blood gas sampling *				
pH	7.36 [7.30–7.44]	7.38 [7.32–7.44]	7.35 [7.27–7.44]	0.152
PCO_2_ (mmHg)	38 [31–49]	39 [32–51]	38 [31–47]	0.331
PO_2_ (mmHg)	71 [61–87]	68 [58–83]	75 [65–95]	0.062
HCO_3_ (mEq/L)	22.5 [18.6–27.5]	24.9 [20–28.5]	20.8 [17.7–25.7]	**0.004**
Lactate (mmol/L)	1.7 [1.2–2.5]	1.4 [1.0–2.1]	2.0 [1.4–2.9]	**<0.001**
WBC (×10^3^/µL)	10.7 [7.5–17.7]	10.3 [7.6–14.5]	12.1 [7.2–16.0]	0.335
NLR	10.6 [5.8–21.2]	10.6 [5.6–20.5]	10.6 [5.9–22.5]	0.488
C-reactive protein (mg/L)	98 [38–169]	78 [27–146]	122 [66–185]	**<0.001**
Procalcitonin (ng/mL)	0.73 [0.26–2.84]	0.45 [0.17–2.12]	0.97 [0.33–5.2]	**<0.001**

* median [25th percentile-75th percentile]. proBNP: Pro–Brain-type Natriuretic Peptide, PCO_2_: Partial Pressure of Carbon Dioxide, PO_2_: Partial Pressure of Oxygen, HCO_3_: Bicarbonate, WBC: White Blood Cell Count, NLR: Neutrophil-to-Lymphocyte Ratio.

**Table 6:** Multivariate analysis for independent risk factors of the requirement of invasive mechanical ventilation in elderly critically ill patients with acute respiratory failure.

	Adjusted OR [95% CI]	*p*
Age	0.996 [0.948–1.005]	0.873
Gender	1.445 [0.657–3.177]	0.360
Pneumonia as a reason for respiratory failure	2.575 [1.083–6.120]	**0.032**
Malignancy as a reason for respiratory failure	2.268 [0.521–9.873]	0.275
P/F ratio	1.001 [0.996–1.005]	0.770
ROX index	0.908 [0.839–0.983]	**0.017**
Pneumonia due to viral etiologies	2.470 [0.419–14.571]	0.318
No pathogen isolated for pneumonia	1.852 [0.713–4.809]	0.206
CRP	0.999 [0.996–1.003]	0.658
Procalcitonin	1.010 [0.996–1.024]	0.156
Length of hospital stay before ICU	1.044 [0.998–1.092]	0.063
Hypoalbuminemia	4.115 [1.743–9.719]	**0.001**

P/F: Partial Pressure of Arterial Oxygen to Fraction of Inspired Oxygen, ROX: Respiratory Rate–Oxygenation Index, CRP: C-reactive Protein, ICU: Intensive Care Unit.

## Data Availability

The dataset is available on request from the authors due to restrictions.

## Abbreviations

The following abbreviations are used in this manuscript:

ARF	Acute Respiratory Failure
ICU	Intensive Care Unit
IMV	Invasive Mechanical Ventilation
SOFA	Sequential Organ Failure Assessment
RRT	Renal Replacement Therapy
APACHE II	Acute Physiology and Chronic Health Evaluation II
RIFLE	Risk, Injury, Failure, Loss, And End-Stage Renal Disease Classification
CHF	Chronic Heart Failure
GCS	Glasgow Coma Scale
SpO_2_	Peripheral Oxygen Saturation
FiO_2_	Fraction of Inspired Oxygen
P/F ratio	The Ratio of Partial Arterial Oxygen Pressure to FiO_2_
ROX	The Respiratory Rate–Oxygenation Index
CRP	C-Reactive Protein
NIV	Non-Invasive Ventilation
HFNO	High-Flow Nasal Oxygen
SD	Standard Deviation
OR	Odds Ratio
COPD	Chronic Obstructive Pulmonary Disease
COVID-19	Coronavirus Disease-2019

## References

[1] He W., Goodkind D., Kowal P. (2016). An Aging World: 2015.

[2] Bagshaw S.M., Webb S.A., Delaney A., George C., Pilcher D., Hart G.K., Bellomo R. (2009). Very old patients admitted to intensive care in Australia and New Zealand: A multi-centre cohort analysis. Crit. Care.

[3] Fuchs L., Novack V., McLennan S., Celi L.A., Baumfeld Y., Park S., Howell M.D., Talmor D.S. (2014). Trends in severity of illness on ICU admission and mortality among the elderly. PLoS ONE.

[4] Fimognari F.L., Lelli D., Landi F., Antonelli Incalzi R. (2022). Association of age with emergency department visits and hospital admissions: A nationwide study. Geriatr. Gerontol. Int..

[5] Behrendt C.E. (2000). Acute respiratory failure in the United States: Incidence and 31-day survival. Chest.

[6] Laporte L., Hermetet C., Jouan Y., Gaborit C., Rouve E., Shea K.M., Si-Tahar M., Dequin P.-F., Grammatico-Guillon L., Guillon A. (2018). Ten-year trends in intensive care admissions for respiratory infections in the elderly. Ann. Intensive Care.

[7] Hsu C.H., Hung Y.M., Chu K.A., Chen C.F., Yin C.H., Lee C.C. (2020). Prognostic nomogram for elderly patients with acute respiratory failure receiving invasive mechanical ventilation: A nationwide population-based cohort study in Taiwan. Sci. Rep..

[8] Vallet H., Schwarz G.L., Flaatten H., de Lange D.W., Guidet B., Dechartres A. (2021). Mortality of Older Patients Admitted to an ICU: A Systematic Review. Crit. Care Med..

[9] Guidet B., Flaatten H., Boumendil A., Morandi A., Andersen F.H., Artigas A., Bertolini G., Cecconi M., Christensen S., Faraldi L. (2022). Withholding or withdrawing of life-sustaining therapy in older adults (≥80 years) admitted to the ICU: A prospective multicenter study. Ann. Intensive Care.

[10] Bonny V., Ladoire M., Gabarre P., Missri L., Urbina T., Guidet B. (2022). Acute respiratory failure in elderly patients. Rev. Prat..

[11] Delerme S., Ray P. (2008). Acute respiratory failure in the elderly. Age Ageing.

[12] Lowery E.M., Brubaker A.L., Kuhlmann E., Kovacs E.J. (2013). The aging lung. Clin. Interv. Aging.

[13] Vincent J.L., Creteur J. (2022). Appropriate care for the elderly in the ICU. J. Intern. Med..

[14] Vincent J.L., Moreno R. (2010). Clinical review: Scoring systems in the critically ill. Crit. Care.

[15] Rodriguez Lima D.R., Anzueta Duarte J.H., Rubio Ramos C., Otálora González L., Pinilla Rojas D.I., Gómez Cortés L.A., Rodríguez Aparicio E.E., Yepes Velasco A.F., Devia Jaramillo G. (2024). Risk factors for in-hospital mortality in older patients with acute respiratory distress syndrome due to COVID-19: A retrospective cohort study. BMC Geriatr..

[16] Hazar A., Gundogus B. (2020). Assessment of inflammatory markers, disease severity and comorbidities in very elderly patients with acute respiratory diseases. Arch. Med. Sci..

[17] Oruç Ö., Morali T., Karakurt Z., Öztas S., Gündogus B. (2016). Hospitalization and mortality rates in patients with respiratory diseases in the very elderly population. J. Gerontol. Geriatr. Res..

[18] Santa Cruz R., Villarejo F., Figueroa A., Cortés-Jofré M., Gagliardi J., Navarrete M. (2019). Mortality in Critically Ill Elderly Individuals Receiving Mechanical Ventilation. Respir. Care.

[19] Reyes L.F., Bastidas Goyes A., Tuta Quintero E.A., Pedreros K.D., Mantilla Y.F., Herrera M., Carmona G.A., Saza L.D., Bello L.E., Muñoz C.A. (2022). Validity of the ROX index in predicting invasive mechanical ventilation requirement in pneumonia. BMJ Open Respir. Res..

[20] Fimognari F.L., De Vincentis A., Arone A., Baffa Bellucci F., Ricchio R., Antonelli Incalzi R. (2024). Clinical outcomes and phenotypes of respiratory failure in older subjects admitted to an acute care geriatric hospital ward. Intern. Emerg. Med..

[21] Ray P., Birolleau S., Lefort Y., Becquemin M.H., Beigelman C., Isnard R., Teixeira A., Arthaud M., Riou B., Boddaert J. (2006). Acute respiratory failure in the elderly: Etiology, emergency diagnosis and prognosis. Crit. Care.

[22] Polok K., Fronczek J., Guidet B., Artigas A., De Lange D.W., Fjølner J., Leaver S., Beil M., Sviri S., Bruno R.R. (2023). Outcomes of patients aged ≥80 years with respiratory failure initially treated with non-invasive ventilation in European intensive care units before and during COVID-19 pandemic. Ann. Intensive Care.

[23] Riquelme R., Torres A., el-Ebiary M Mensa J., Estruch R., Ruiz M., Angrill J., Soler N. (1997). Community-acquired pneumonia in the elderly. Clinical and nutritional aspects. Am. J. Respir. Crit. Care Med..

[24] Qiao Q., Lu G., Li M., Shen Y., Xu D. (2012). Prediction of outcome in critically ill elderly patients using APACHE II and SOFA scores. J. Int. Med. Res..

[25] Erdogan E., Findikli H.A. (2022). The prognostic value of serum albumin levels on mortality in elderly patients with sepsis. Eur. Geriatr. Med..

[26] Dündar N.B., İnci K., Hasmercan B., Türkoğlu M., Aygencel G. (2025). The Influence of Hypophosphatemia on ICU Outcomes in Elderly Critically Ill Patients. Eur. J. Geriatr. Gerontol..

[27] Corona L.P., de Oliveira Duarte Y.A., Lebrão M.L. (2018). Markers of nutritional status and mortality in older adults: The role of anemia and hypoalbuminemia. Geriatr. Gerontol. Int..

[28] Yin M., Si L., Qin W., Li C., Zhang J., Yang H., Han H., Zhang F., Ding S., Zhou M. (2018). Predictive Value of Serum Albumin Level for the Prognosis of Severe Sepsis Without Exogenous Human Albumin Administration: A Prospective Cohort Study. J. Intensive Care Med..

[29] Farfel J.M., Franca S.A., Sitta M.C., Jacob Filho W., Carvalho C.R. (2009). Age, invasive ventilatory support and outcomes in elderly patients admitted to intensive care units. Age Ageing.

[30] Flaatten H., De Lange D.W., Artigas A., Bin D., Moreno R., Christensen S., Joynt G.M., Bagshaw S.M., Sprung C.L., Benoit D. (2017). The status of intensive care medicine research and a future agenda for very old patients in the ICU. Intensive Care Med..

[31] Vo-Pham-Minh T., Duong-Thi-Thanh V., Nguyen T., Phan-Tran-Xuan Q., Phan-Thi H., Bui-Anh T., Duong-Thien P., Duong-Quy S. (2021). The impact of risk factors on treatment outcomes of nosocomial pneumonia due to Gram-negative bacteria in the intensive care unit. Pulm. Ther..

[32] Hope A.A., Adeoye O., Chuang E.H., Hsieh S.J., Gershengorn H.B., Gong M.N. (2018). Pre-hospital frailty and hospital outcomes in adults with acute respiratory failure requiring mechanical ventilation. J. Crit. Care.

[33] Roca O., Messika J., Caralt B., García-de-Acilu M., Sztrymf B., Ricard J.D., Masclans J.R. (2016). Predicting success of high-flow nasal cannula in pneumonia patients with hypoxemic respiratory failure: The utility of the ROX index. J. Crit. Care.

[34] Roca O., Caralt B., Messika J., Samper M., Sztrymf B., Hernández G., García-de-Acilu M., Frat J.P., Masclans J.R., Ricard J.D. (2019). An Index Combining Respiratory Rate and Oxygenation to Predict Outcome of Nasal High-Flow Therapy. Am. J. Respir. Crit. Care Med..

[35] Artacho Ruiz R., Artacho Jurado B., Caballero Güeto F., Cano Yuste A., Durbán García I., García Delgado F., Guzmán Pérez J.A., López Obispo M., Quero Del Río I., Rivera Espinar F. (2021). Predictors of success of high-flow nasal cannula in the treatment of acute hypoxemic respiratory failure. Med. Intensiv..

[36] Zhou F., Yu T., Du R., Fan G., Liu Y., Liu Z., Xiang J., Wang Y., Song B., Gu X. (2020). Clinical course and risk factors for mortality in COVID-19 ICU patients. Lancet.

[37] Ugajin M., Yamaki K., Iwamura N., Yagi T., Asano T. (2012). Blood urea nitrogen to serum albumin ratio independently predicts mortality and severity of community-acquired pneumonia. Int. J. Gen. Med..

